# Complete genome sequence of *Pseudomonas aeruginosa* bacteriophage PaFZ4 isolated from Dhaka, Bangladesh

**DOI:** 10.1128/mra.00993-25

**Published:** 2025-10-31

**Authors:** Zuhayr Mahtab, Fahmida Haque Tamanna, Ishrat Jabeen, Sohidul Islam, Sezanur Rahman, Mustafizur Rahman, Sabbir R. Shuvo

**Affiliations:** 1 Department of Biochemistry & Microbiology, North South University 54495, Dhaka, Bangladesh; 2 Virology Laboratory, Infectious Diseases Division, icddr,b 56291 , Dhaka, Bangladesh; Department of Biology, Queens College, Queens, New York, USA

**Keywords:** Bacteriophages, *Pseudomonas aeruginosa*, phage genomics, whole-genome sequencing, *Phikmvvirus*, Bangladesh

## Abstract

Here, we report the complete genome sequence of bacteriophage PaFZ4, a lytic bacteriophage infecting *Pseudomonas aeruginosa* strains. The PaFZ4 was isolated from hospital wastewater in Dhaka, Bangladesh, and predicted to be under the genus *Phikmvvirus* (family *Autographiviridae*). The genome of PaFZ4 has a 43,335-bp linear genome with 65 coding sequences.

## ANNOUNCEMENT

*Pseudomonas aeruginosa* is a gram-negative, bacillus-shaped bacterium about 1–5 µm long and 0.5–1.0 µm wide (1). Drug-resistant *P. aeruginosa* causes serious public health problems and is listed as an ESKAPE pathogen group by the World Health Organization (2). The main objective of this study was to perform genomic analysis of bacteriophage PaFZ4, a lytic phage isolated from hospital sewage.

Sewage samples (250 mL each) were collected on 31 May 2023 from Kurmitola General Hospital, Dhaka, Bangladesh. The samples were filtered using a 0.45 µm Whatman filter paper and a 0.22 µm syringe filter to remove large debris and bacteria. Subsequently, centrifugation (CT 15RE, Hitachi, Japan) was carried out at 4°C at 12,000 rpm for 15 min. The sample was then filtered again through a 0.22 µm filter and transferred to a fresh Eppendorf tube (3).

The strains of *P. aeruginosa* were isolated from various sewage samples collected around Bashundhara R/A, Dhaka, by serial dilution and cultivation on cetrimide agar (Himedia, India). Pure colonies of *P. aeruginosa* were isolated from the cetrimide plates. Later, processed filter sewage samples were added with 200 µL of *P. aeruginosa* (OD_600_ of 0.5, 150 rpm at 37°C) at different ratios (1:1 and 1:2) and plated on LB for the double-layer agar method (4). Plates were incubated at 37°C for 24 h to visualize the formation of plaques. Individual plaques were purified through three successive rounds of single-plaque isolation. For the whole-genome sequencing, DNA was extracted from a single plaque-purified lysate using the DNeasy Blood & Tissue Kit protocol (QIAGEN, USA) (5).

**Fig 1:**
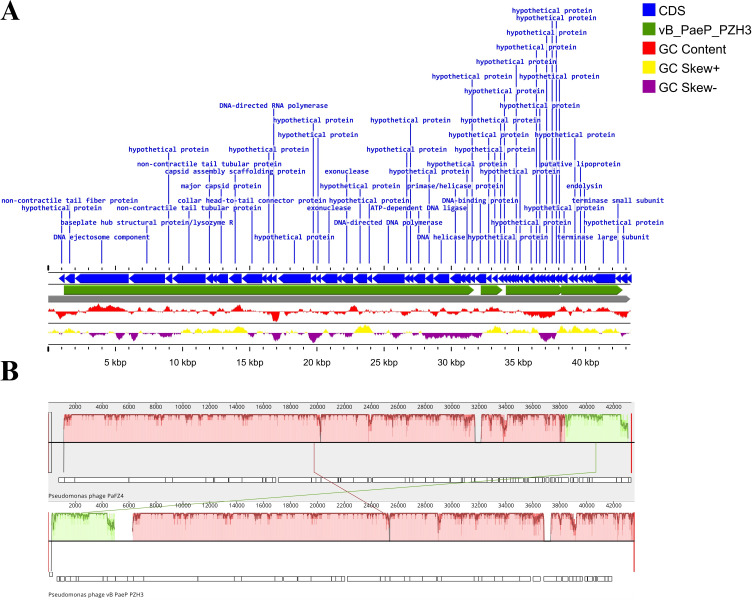
(**A**) Sequence alignment of the isolated PaFZ4 phage with *P. aeruginosa* phage vB_PaeP_PZH3 (accession no. PQ562891). The gaps in each genome represent the missing regions between PaFZ4 and the reference. The gray color represents the PaFZ4 sequence, and the green indicates the reference genome of Phage vB_PaeP_PZH3. The blue arrows denote the annotated coding DNA sequences (CDS). The yellow peaks correspond to the positive GC skew, while the purple peaks represent the negative GC skew. Red peaks indicate the GC content. (**B**) Whole-genome sequence alignment of PaFZ4 and vB_PaeP_PZH3 using Mauve. Locally collinear blocks (LCBs) indicate conserved regions; each sequence of identically colored blocks represents a collinear set of matching regions. The high level of synteny reflects evolutionary conservation between the two phages.

The DNA library was prepared using Illumina DNA Prep (Illumina, San Diego, CA, USA) and sequenced on the Illumina MiSeq platform with 500 cycles, yielding 698,258 paired-end reads (2 × 250 bp) with an average read length of 250 bp. Raw reads were processed on Galaxy Europe (https://usegalaxy.eu/): quality control with FastQC v0.11.2 (6), adapter trimming with Trimmomatic v0.39 (7), *De novo* assembly using SPAdes v4.2.0 (8), and polishing with Pilon v1.20 (9). Coverage depth was calculated using BBMap v35.85 (10). Assembly assessment was checked with Quast v5.3 (11), and genome completeness (100%) was confirmed with CheckV v1.0.3 (12). The length of the assembled genome was 43,335 bp, with 62.31% GC content (Table 1; Fig. 1). The topology of the genome was confirmed to be linear double-stranded DNA using Bandage (13). Genome termini were analyzed using PhageTerm (14), which identified a headful (pac-type) packaging mechanism.

**TABLE 1:** Isolation source, sequencing details, and genomic features of phage PaFZ4

Attribute	PaFZ4
Place of isolation	Dhaka, Bangladesh
Isolation source	Hospital wastewater
Genome length (bp)	43,335
GC%	62.31
No. of CDS	65
Coverage	645.16
Family	*Autographiviridae*
Genus	*Phikmvvirus*
NCBI accession number	PV610699

Annotations performed with PHANOTATE 1.5.0 (15) in BV-BRC (https://www.bv-brc.org/), with Bacteriophages as the annotation recipe, identified 65 coding sequences, with 21 having putative functions, and 44 hypothetical proteins. Sequence alignment genomic maps of PaFZ4 against *P. aeruginosa* phage vB_PaeP_PZH3 (accession no. PQ562891) were generated using Proksee (16) and Mauve (17) (Fig. 1). Phage vB_PaeP_PZH3 was selected as a reference genome due to the highest BLASTn similarity (96.94% identity and 94% coverage). The lifestyle of PaFZ4 was predicted to be virulent (lytic) using PhageAI Life Cycle Classifier v1.6.0 (18). PhaBOX (19) was used to find the taxonomic group of PaFZ4. Default parameters were used for all the programs unless otherwise mentioned.

## Data Availability

The complete genome sequence of phage PaFZ4 has been deposited in the NCBI GenBank under accession number PV610699 and in the Sequence Read Archive under accession number PRJNA1320931.
